# Oral Cavity Cancer and the Subsequent Incidence of Other Cancer Diagnoses: A Retrospective Cohort Study in German General Practices

**DOI:** 10.3390/cancers18213444

**Published:** 2026-10-24

**Authors:** Lisa Lotta Cirkel, Bernd Wöstmann, Karel Kostev

**Affiliations:** 1 Department of Cariology, Periodontology, and Endodontology, Dental Clinic, Justus Liebig University Giessen, 35392 Giessen, Germanybernd.woestmann@dentist.med.uni-giessen.de (B.W.); 2 Department of Prosthodontics, Dental Clinic, Justus Liebig University Giessen, 35392 Giessen, Germany; 3 University of Marburg, University Hospital, 35043 Marburg, Germany; 4 Epidemiology, IQVIA, 60549 Frankfurt am Main, Germany

**Keywords:** oral cavity cancer, second primary cancer, primary care, retrospective cohort study, propensity score matching, Germany

## Abstract

Background/Objectives: Subsequent cancer diagnoses are well documented after oral cavity cancer, particularly within the upper aerodigestive tract. However, evidence from routine primary care is limited, although such data may provide complementary insights into the long-term documentation of subsequent cancer diagnoses. This study examined subsequent cancer diagnoses after oral cavity cancer in German general practices and assessed the influence of anatomically adjacent cancer codes. Methods: This retrospective cohort study used the IQVIA Disease Analyzer database. Adults with a first documented oral cavity cancer (International Classification of Diseases, 10th Revision [ICD-10]: C02–C06) recorded in German general practices between 2005 and 2024, with at least 12 months of prior observation and no earlier cancer, were included. A landmark design was applied to both cohorts: follow-up began 365 days after the index date, and individuals not observed beyond that date or with a cancer documented in the first year were excluded. Patients were matched 1:10 on age, sex, index year, consultation frequency in the 12 months before the index date and Charlson Comorbidity Index. The outcome definition was identical in both cohorts (ICD-10 C00–C97, excluding C02–C06 and C77–C80). Cox regression with robust variance estimation was used. Results: A total of 1043 patients with oral cavity cancer and 10,430 matched individuals were analyzed. A cancer diagnosis was documented in 16.8% and 6.9% of individuals, respectively, corresponding to 38.0 and 15.6 per 1000 person-years (hazard ratio [HR] 2.43; 95% confidence interval [CI] 2.06–2.87). The HR was highest among patients aged ≤60 years (HR 4.07) and lowest among those aged >70 years (HR 1.48; *p* for interaction <0.001). Subsequent diagnoses involving the upper aerodigestive and respiratory tracts were disproportionately represented in the oral cavity cancer cohort. Excluding anatomically adjacent cancer codes attenuated the association (HR 1.81; 95% CI 1.51–2.17). Conclusions: Oral cavity cancer was associated with a higher incidence of subsequently documented cancer diagnoses. The association was attenuated but persisted after excluding anatomically adjacent and potentially ambiguous cancer codes; the data do not establish whether these diagnoses represent confirmed second primary malignancies.

## Simple Summary

People who have had cancer of the mouth are known to be at higher risk of further cancers, especially in the throat and lungs. Most of this knowledge comes from cancer registries and specialist hospitals. We used records from more than 1000 German general practices to compare 1043 patients with mouth cancer with 10,430 people of similar age, sex and health status who did not have mouth cancer. In the following years, the rate of subsequently documented cancer diagnoses was higher in patients with mouth cancer (38.0 versus 15.6 per 1000 person-years), most notably in the head and neck region and in the airways and lungs. Some of these records may describe the original tumor rather than a new cancer, but a clear difference remained after such diagnoses were set aside. Family doctors should therefore remain attentive to new cancers during the long-term care of patients who have had mouth cancer.

## 1. Introduction

Cancers of the lip and oral cavity represent a substantial global health burden, with approximately 389,500 new cases and 188,200 deaths estimated worldwide in 2022 [1].

Most malignant tumors of the oral cavity are squamous cell carcinomas, and tobacco and alcohol consumption are among the best-established exposures associated with their development [2,3,4]. These associations are not limited to the oral cavity but have also been observed across several head and neck cancer sites, including the oropharynx and larynx [5].

In patients with oral cavity cancer, subsequent malignancies have repeatedly been documented, particularly in the head and neck region and the respiratory tract. This has been described in population-based studies from Finland, Denmark, and Korea [6,7,8]. One biological concept proposed to explain this pattern is field cancerization, originally described by Slaughter et al. [9]. It refers to the presence of histologically or molecularly altered mucosal areas extending beyond the clinically apparent primary tumor; such areas may contribute to the development of additional malignancies within the same region [9,10]. At the same time, previous studies indicate that the occurrence of subsequent malignancies may vary according to patient age, anatomical site, and follow-up period. For example, the Korean registry study reported significant differences according to age and the location of the original oral cavity carcinoma [8].

The temporal distribution of subsequent cancer-related events is also relevant when interpreting long-term outcomes. Locoregional recurrences occur predominantly during the first years after treatment, whereas second primary tumors remain clinically relevant over longer follow-up periods [11,12]. Lung cancer is of particular relevance in this context, as it has been frequently documented among survivors of head and neck cancer [13,14].

Despite these findings, several important gaps remain. First, most available evidence derives from cancer registries or specialized head and neck cancer cohorts, with comparisons commonly made against the general population rather than matched individuals with similar demographic and clinical characteristics. Second, evidence from routine primary care is limited, although general practitioners may remain involved in long-term follow-up and document newly diagnosed malignancies over many years. German primary care data have been used to investigate subsequent cancer diagnoses in other patient populations, but, to our knowledge, not after oral cavity cancer [15,16,17]. Third, routine data do not reliably distinguish a true metachronous second primary tumor from recurrence, regional extension, or recoding of the index tumor, particularly for anatomically adjacent sites.

The aim of this study was therefore to examine the incidence of cancer diagnoses other than the index tumor in patients with oral cavity cancer documented in German general practices, in comparison with matched individuals without oral cavity cancer, and to assess the extent to which the observed association depends on the handling of codes that are anatomically adjacent to the index tumor.

## 2. Materials and Methods

### 2.1. Database

This retrospective cohort study was based on data from the IQVIA Disease Analyzer database, which contains anonymized data on diagnoses, prescriptions and basic medical and demographic characteristics obtained directly from the practice computer systems of office-based physicians in Germany [18]. The database has been used in several previous studies of cancer epidemiology [15,16,17]. The sampling method for the Disease Analyzer database uses statistics from the German Medical Association to determine the panel design according to specialist group, German federal state, community size category and age of physician, and the panel of practices has been shown to be representative of general and specialized practices in Germany [18]. The present analysis was restricted to general practices; practices with a diabetological focus were excluded. Data were available from January 2000 to February 2026.

### 2.2. Study Population

**Figure 1:**
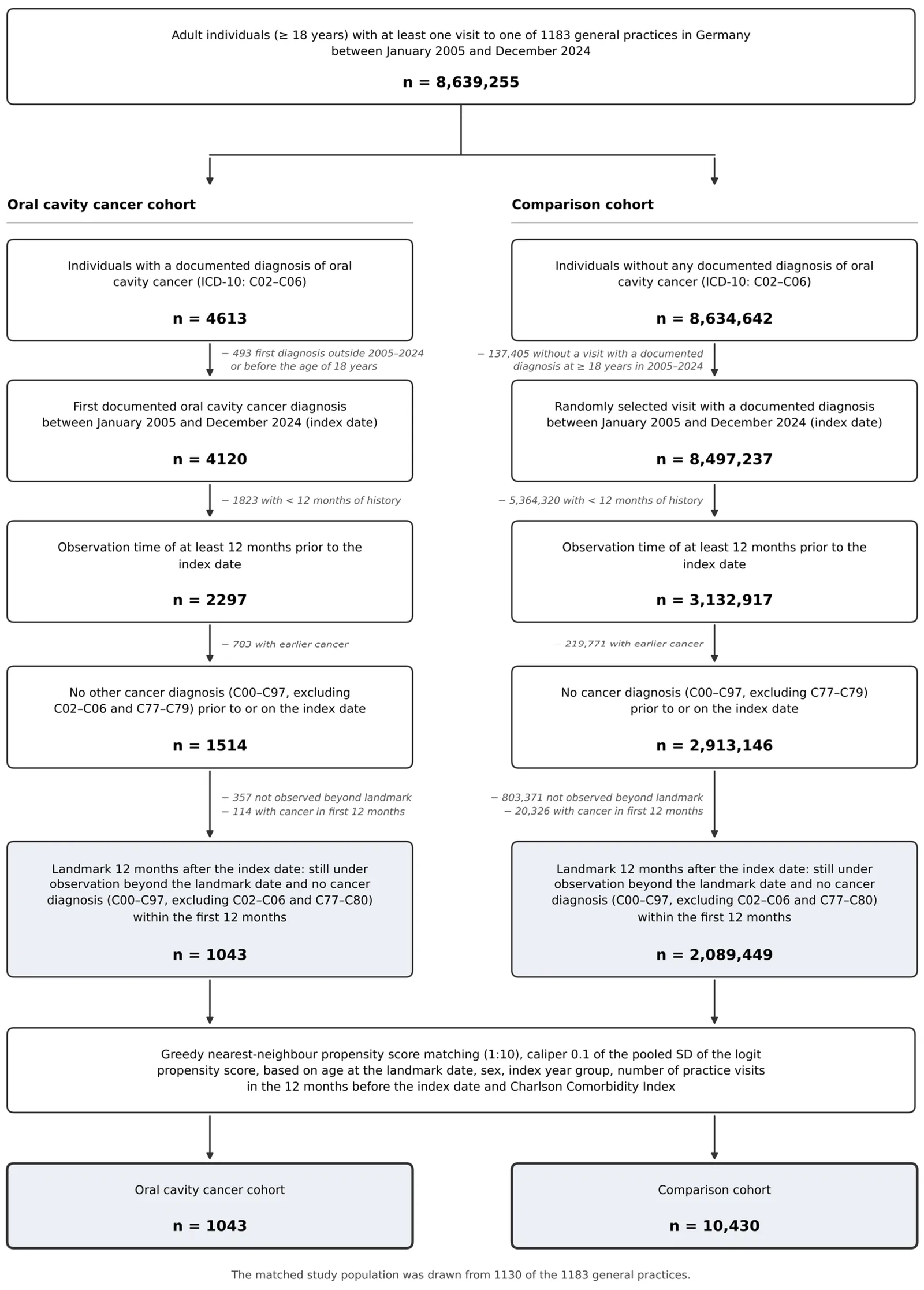
Selection of the study population.

The study included patients aged 18 years or older with a first documented diagnosis of oral cavity cancer—tongue, other and unspecified parts (International Classification of Diseases, 10th Revision [ICD-10]: C02), gum (C03), floor of mouth (C04), palate (C05), and other and unspecified parts of mouth (C06)—recorded in one of 1183 general practices in Germany between January 2005 and December 2024. The date of the first documented oral cavity cancer diagnosis was defined as the index date. Four-digit ICD-10 codes were available (Supplementary Table S5); in 20 patients (1.9%) with two different oral cavity sites coded on the index date, the first recorded code determined the index site. Diagnoses documented by general practitioners may also have been made previously by specialists in other practices or in hospitals. A further inclusion criterion was an observation time of at least 12 months prior to the index date. Patients with any other cancer diagnosis (ICD-10: C00–C97, excluding C02–C06 and the secondary neoplasm codes C77–C79) documented prior to or on the index date were excluded, including oropharyngeal codes, leaving 1514 patients (Figure 1).

Individuals without any documented diagnosis of oral cavity cancer served as the comparison cohort. For this cohort, the index date was the date of a randomly selected practice visit with a documented diagnosis between January 2005 and December 2024. The same requirements of an age of at least 18 years and an observation time of at least 12 months prior to the index date were applied, and individuals with any cancer diagnosis (C00–C97, excluding C77–C79) prior to or on the index date were excluded, leaving 2,913,146 individuals (Figure 1).

A landmark design was applied to both cohorts [19]. Follow-up started 365 days after the index date, and eligibility was assessed at that landmark: individuals had to remain under observation in the practice beyond the landmark date, and individuals with a cancer diagnosis meeting the outcome definition (Section 2.3) during the first 12 months after the index date were excluded. Repeated documentation of the index tumor (C02–C06) and codes C77–C80 therefore did not lead to exclusion, and the same criteria were applied in both cohorts. This latency period was applied to reduce the influence of synchronous tumors and cancer codes arising from the diagnostic work-up of the index tumor, which are expected to be concentrated early after the index diagnosis. Applying the landmark left 1043 of the 1514 patients with oral cavity cancer (68.9%) and 2,089,449 of the 2,913,146 individuals without oral cavity cancer (71.7%); the numbers excluded for each criterion are shown in Figure 1.

Individuals without oral cavity cancer were then matched to patients with oral cavity cancer using greedy nearest-neighbor propensity score matching in a 1:10 ratio, with a caliper of 0.1 pooled standard deviations of the logit of the propensity score. The propensity score model included age at the landmark date, sex, index year group, the number of practice visits in the 12 months before the index date and the Charlson Comorbidity Index (CCI, excluding the cancer and metastatic solid tumor components, calculated from all diagnoses documented up to the landmark date, so that all matching variables refer to the start of follow-up) [20]. Visit frequency during follow-up was not used because it is measured after the index date and may be influenced by both the exposure and the outcome [21,22]. Matching yielded 1043 matched sets comprising 1043 patients with oral cavity cancer and 10,430 matched individuals without oral cavity cancer, all of whom contributed follow-up time to the analyses. The matched study population was drawn from 1130 of the 1183 general practices (Figure 1).

### 2.3. Study Outcomes and Statistical Analyses

The outcome of the study was the first documented diagnosis of a cancer other than the index tumor. The outcome definition was identical in both cohorts: ICD-10 C00–C97, excluding C02–C06, C77–C79 and C80 (malignant neoplasm without specification of site). Code C80 was excluded because in patients with a known tumor this code cannot be distinguished from a recoding or dissemination of the index tumor. Because individuals with a documented oral cavity cancer were not eligible for the comparison cohort, C02–C06 did not occur as outcomes in either cohort. Codes not counted as outcomes did not end follow-up; individuals remained under observation for a later eligible diagnosis. Follow-up ran from the landmark date to the first documented cancer diagnosis or to the date of the last documented practice visit, whichever occurred first; the administrative end of follow-up was February 2026.

Baseline characteristics of the two cohorts were compared using standardized mean differences (SMD), with values below 0.10 taken to indicate acceptable balance. The cumulative incidence of a cancer diagnosis was estimated using the Kaplan–Meier method and is displayed for the first five years of follow-up, after which the number at risk declined markedly; the curves for the two cohorts were compared using the log-rank test. Incidence rates per 1000 person-years and Cox proportional hazards regression models were based on the entire available follow-up, with robust (sandwich) standard errors accounting for the matched sets [23,24]. The proportional hazards assumption was assessed using Schoenfeld residuals, a time-by-exposure interaction term and HRs estimated separately for 0–1, >1–3 and >3 years after the landmark date [25]. Results of the Cox models are reported as hazard ratios (HR) with 95% confidence intervals (CI). The overall model and the models for the pre-specified subgroups—three age groups (≤ 60, 61–70 and > 70 years) and the two sexes—were regarded as the primary analyses. For these six models, a Bonferroni-corrected significance threshold of 0.05/6 = 0.008 was applied. Differences between age groups were tested using an exposure-by-age-group interaction term, and differences between sexes by comparing the sex-specific HRs. The analyses by site of the oral cavity cancer and sensitivity analyses were regarded as exploratory and were not included in the correction.

In a pre-specified sensitivity analysis, cancers of the lip, oral cavity and pharynx (C00–C14), of bone and articular cartilage (C41) and of other and ill-defined sites (C76) were additionally not counted as outcomes in either cohort, because these sites are anatomically adjacent to or overlap with the index tumor. The matched cohort was left unchanged for this analysis, so that only the outcome definition varied. Further sensitivity analyses are described in Supplementary Table S2; they included restriction to patients with at least three years of prior observation and a 24-month landmark, to address a possible delay between the actual diagnosis and its documentation in general practice, and matching on a CCI calculated only from diagnoses documented before the index date. The distribution of the time to the documented cancer diagnosis was compared between head and neck outcomes and all other sites within the oral cavity cancer cohort using the Wilcoxon rank-sum test and a chi-squared test for the proportion of events occurring in the first year after the landmark. Analyses were carried out using SAS version 9.4 (SAS Institute, Cary, NC, USA).

## 3. Results

### 3.1. Baseline Characteristics of the Study Sample

The study included 1043 patients with oral cavity cancer and 10,430 matched individuals without oral cavity cancer (Table 1). Because of the matching procedure, the two cohorts were closely comparable. The mean age was 63.5 years (standard deviation [SD] 13.3) and 63.6 years (SD 13.5); 61.9% and 61.2% were men, and the median Charlson Comorbidity Index was 2 in both cohorts. The median number of practice visits in the 12 months before the index date was 5 (interquartile range [IQR] 1–11) in both cohorts, and all standardized mean differences were below 0.10; this also applied to the CCI calculated from diagnoses documented before the index date (SMD 0.030). Within the oral cavity cancer cohort, the most frequently documented sites were tongue, other and unspecified parts (C02; 374 patients, 35.9%) and floor of mouth (C04; 318 patients, 30.5%), followed by other and unspecified parts of mouth (C06; 227 patients, 21.8%), palate (C05; 76 patients, 7.3%) and gum (C03; 48 patients, 4.6%).

### 3.2. Association Between Oral Cavity Cancer and Subsequent Cancer Diagnoses

**Figure 2:**
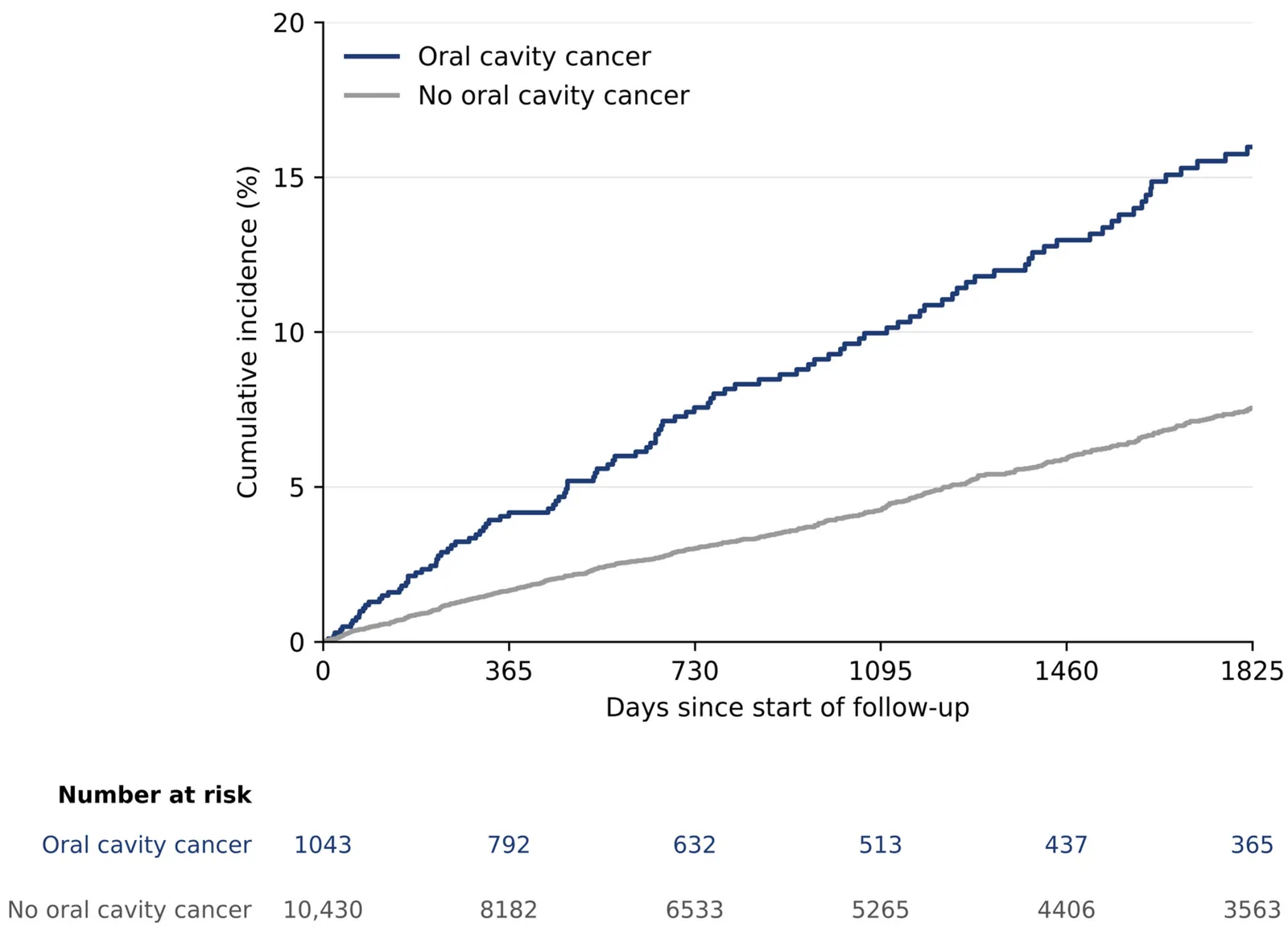
Cumulative incidence of a documented cancer diagnosis over the first five years after the landmark date in patients with oral cavity cancer and matched individuals without oral cavity cancer (Kaplan–Meier estimates, with numbers at risk; the estimates assume non-informative censoring at the last practice visit and do not account for death as a competing event).

A cancer diagnosis was documented during follow-up in 175 of the 1043 patients with oral cavity cancer (16.8%) and in 722 of the 10,430 matched individuals (6.9%), with a median follow-up of 2.9 (IQR 1.1–6.9) and 3.0 (IQR 1.2–6.6) years and 4609 and 46,337 person-years, respectively. The cumulative incidence of a cancer diagnosis was higher in the oral cavity cancer cohort than in the matched cohort from the beginning of follow-up onwards, and the two curves diverged progressively (log-rank χ^2^ = 118.7; *p* < 0.001; Figure 2). The Kaplan–Meier estimates of the cumulative incidence were 4.2% in the oral cavity cancer cohort and 1.6% in the matched cohort one year after the landmark date; the corresponding figures were 7.6% and 3.0% after two years, 10.0% and 4.2% after three years, and 16.0% and 7.5% after five years. The absolute difference between the two cohorts widened from 2.6 percentage points at one year to 8.4 percentage points at five years. These estimates assume that censoring at the last practice visit is non-informative and do not account for death as a competing event; the proportions of individuals still under observation were similar in both cohorts (41.5% and 36.9% at five years; Supplementary Table S4). Over the entire follow-up, the incidence rate was 38.0 per 1000 person-years in the oral cavity cancer cohort and 15.6 per 1000 person-years in the matched cohort (Table 2).

In the Cox regression analysis, oral cavity cancer was associated with the subsequent documentation of another cancer diagnosis (HR 2.43; 95% CI 2.06–2.87; *p* < 0.001; Table 2). There was no evidence of non-proportional hazards (Supplementary Table S3). The point estimates varied across age groups (*p* for interaction < 0.001). The HR was highest among patients aged ≤60 years (HR 4.07; 95% CI 3.00–5.51; *p* < 0.001), followed by patients aged 61–70 years (HR 2.57; 95% CI 1.98–3.33; *p* < 0.001), and was lowest among patients aged >70 years (HR 1.48; 95% CI 1.08–2.03; *p* = 0.015). The latter association did not meet the Bonferroni-corrected significance threshold. This pattern coincided with a rise in the incidence rate in the comparison cohort from 7.6 to 27.4 per 1000 person-years across the three age groups, whereas the rate in the oral cavity cancer cohort remained between 31.4 and 46.7 per 1000 person-years. The point estimate was numerically higher in men (HR 2.62; 95% CI 2.13–3.22; *p* < 0.001) than in women (HR 2.13; 95% CI 1.61–2.82; *p* < 0.001), but the difference was not statistically significant (*p* = 0.24). The incidence rate was also higher among men with oral cavity cancer than among women with oral cavity cancer (44.7 versus 28.3 per 1000 person-years). Because individuals with an oral cavity cancer recorded at any time were not eligible for the primary comparison cohort, individuals with a later oral cavity cancer were additionally allowed into the comparison cohort and censored at that diagnosis; the HR was 2.35 (95% CI 2.00–2.77). Matching on a CCI based on diagnoses documented before the index date yielded an HR of 2.41 (95% CI 2.04–2.84). The association was consistent across all further sensitivity analyses (HR range 2.35–2.64; Supplementary Table S2).

In the exploratory analyses stratified by the site of the oral cavity cancer, the point estimates were consistent across all five sites, ranging from 1.96 to 2.95, and all five confidence intervals excluded unity (Table 3). The corresponding incidence rates in the oral cavity cancer cohort ranged from 33.2 per 1000 person-years for cancer of the palate to 45.8 for other and unspecified parts of the mouth, while the rates in the matched sub-cohorts varied between 13.6 and 19.2.

### 3.3. Distribution of the Documented Cancer Diagnoses

The distribution of the cancer diagnoses documented during follow-up differed substantially between the two cohorts (Table 4). In the oral cavity cancer cohort, 39 of 175 documented cancers (22.3%) were coded to the lip, oral cavity and pharynx block (C00–C14), compared with fewer than 10 of 722 in the matched cohort; within this block, base of tongue (C01) and oropharynx (C10) accounted for 32 of the 39 diagnoses. Cancers of the respiratory and intrathoracic organs were also more frequent in the oral cavity cancer cohort (17.7% versus 10.7%; lung, C34: 14.9% versus 9.6%); cancers of bone and articular cartilage and of other and ill-defined sites were each documented fewer than 10 times per cohort.

By contrast, the site groups that predominated in the matched cohort accounted for smaller proportions in the oral cavity cancer cohort: male genital organs (8.6% versus 17.2%), digestive organs (14.3% versus 17.7%), other malignant neoplasms of skin (13.7% versus 18.0%) and urinary tract (6.3% versus 9.4%); breast and lymphoid and hematopoietic cancers accounted for 8.2% and 8.0% of diagnoses in the matched cohort and for fewer than 10 diagnoses each in the oral cavity cancer cohort. These percentages describe the composition of the documented diagnoses and are not site-specific incidence estimates. In an exploratory analysis of lung cancer as a separate outcome, the incidence rates were 6.1 and 1.6 per 1000 person-years (HR 3.74; 95% CI 2.43–5.76; Supplementary Table S2).

### 3.4. Sensitivity Analysis and Timing of the Documented Diagnoses

When cancers of the lip, oral cavity and pharynx, of bone and articular cartilage and of other and ill-defined sites were additionally not counted as outcomes, the association was attenuated but remained significant (HR 1.81; 95% CI 1.51–2.17; *p* < 0.001; Supplementary Table S1), with incidence rates of 27.8 and 15.3 per 1000 person-years. The age gradient persisted (*p* for interaction = 0.005), with the association no longer statistically significant in patients older than 70 years (HR 1.20; 95% CI 0.85–1.68; *p* = 0.299).

Within the oral cavity cancer cohort, the timing of the documented diagnoses differed between the two groups of sites. Of the 47 diagnoses coded to the lip, oral cavity and pharynx, to bone and articular cartilage or to other and ill-defined sites, 18 (38.3%) were documented within the first year after the landmark date, compared with 21 of 128 diagnoses (16.4%) at all other sites (*p* = 0.002). Across the whole follow-up period, the distributions of the time to diagnosis did not differ significantly between the two groups (median 2.8 versus 3.8 years; *p* = 0.096).

## 4. Discussion

In this matched cohort study based on German general practice data, patients with oral cavity cancer had a substantially higher incidence of subsequently documented cancer diagnoses than matched individuals without oral cavity cancer. The association was observed in both sexes and across all oral cavity cancer subsites, with the highest point estimate among patients aged 60 years or younger. Importantly, the association was attenuated but remained statistically significant after excluding cancer codes that were anatomically adjacent to or potentially overlapping with the index tumor. However, the data do not allow the remaining diagnoses to be regarded as confirmed independent primary malignancies.

### 4.1. Comparison with Previous Studies

The present findings are broadly consistent with previous population-based studies from Finland, Denmark, and Korea, which reported an elevated occurrence of second primary malignancies after oral cavity cancer that varied according to patient age and the anatomical site of the primary tumor [6,7,8].

Direct comparison of effect estimates should nevertheless be made cautiously because of methodological differences between studies. Previous investigations have predominantly relied on cancer registries or specialized oncology cohorts and have frequently compared patients with oral cavity cancer with the general population [26,27]. In contrast, the present study used a matched comparison cohort balanced for age, sex, index year, comorbidity, and consultation frequency before the index date. On the other hand, the present analysis was based on cancer diagnoses documented in general practice and lacked histopathological verification. The present study therefore evaluates subsequent cancer diagnoses rather than confirmed second primary malignancies.

### 4.2. Age and Sex

The point estimates showed an age-related gradient, with an HR of 4.07 among patients aged ≤60 years, 2.57 among those aged 61–70 years, and 1.48 among those aged >70 years. Similar age-related variation in the occurrence of second primary cancers has previously been reported by Min et al. [8]. A formal interaction test supported a difference between age groups (*p* < 0.001).

The incidence rates provide additional context for this pattern. Within the oral cavity cancer cohort, incidence rates remained relatively similar across age groups, whereas the incidence in the matched comparison cohort increased markedly with age. Thus, the decreasing relative association with increasing age may partly reflect the rising background incidence of cancer rather than a substantial reduction in subsequent cancer diagnoses among older patients.

The point estimate was numerically higher in men than in women, with HRs of 2.62 and 2.13, respectively, but this difference was not statistically significant. In addition, the incidence rate among men with oral cavity cancer was higher than that among women. Differences in exposure to tobacco and alcohol may contribute to this pattern, as both are established risk factors for cancers of the oral cavity and other head and neck sites [5]. However, information on smoking and alcohol consumption was unavailable in the present database, and the observed sex difference therefore cannot be directly attributed to these factors.

### 4.3. Distribution of Subsequent Cancer Diagnoses

The distribution of subsequent cancer diagnoses differed markedly between the two cohorts. In patients with oral cavity cancer, diagnoses involving the lip, oral cavity and pharynx accounted for 22.3% of documented cancers, compared with fewer than 10 of 722 diagnoses in the matched cohort, while respiratory and intrathoracic malignancies accounted for 17.7% and 10.7%, respectively. In contrast, several other cancer groups, including male genital, breast and hematological malignancies, were proportionally less frequent in the oral cavity cancer cohort.

This pattern is compatible with the distribution reported in previous population-based studies, in which second primary cancers after oral cavity cancer occurred particularly often in the head and neck region and the respiratory tract [7,8]. Shared exposure to tobacco and alcohol may contribute to this distribution. Field cancerization may additionally be relevant for the occurrence of multiple malignant lesions within anatomically related mucosal regions of the upper aerodigestive tract [10]. However, the present data do not allow the relative contributions of shared carcinogenic exposure, field cancerization and differences in medical surveillance [28] to be distinguished.

The relatively high proportion of respiratory and intrathoracic malignancies is also consistent with previous evidence showing an increased occurrence of subsequent lung cancer among survivors of head and neck cancer [13,14]. Because malignancies of the respiratory tract are anatomically distinct from the oral cavity, these findings are less likely to reflect coding overlap with the index tumor, although more intensive diagnostic imaging and shared exposure to tobacco may also contribute.

### 4.4. Codes Adjacent to the Index Tumor

Several diagnostic groups require cautious interpretation because of their anatomical proximity to the index tumor. Cancers of bone and articular cartilage and of other ill-defined sites were proportionally more frequent in the oral cavity cancer cohort, and most diagnoses within the lip, oral cavity and pharynx block involved the base of tongue or oropharynx. In the absence of histological information, laterality and tumor stage, these diagnoses cannot be reliably distinguished from reclassification, local recurrence or regional extension of the index tumor. Established criteria for defining a second primary tumor therefore cannot be fully applied to routine primary care data [10]. Clinical studies distinguishing second primary tumors from local recurrences generally rely on additional anatomical, pathological and temporal criteria that are unavailable in the present database [29,30].

The sensitivity analysis was therefore central to the interpretation of the findings. Excluding anatomically adjacent and potentially ambiguous cancer codes reduced the hazard ratio from 2.43 to 1.81 and the incidence rate in the oral cavity cancer cohort from 38.0 to 27.8 per 1000 person-years. This attenuation indicates that such codes contributed substantially to the primary association. The association persisted after exclusion of these code groups; however, the available data cannot determine how much of the remaining association reflects true subsequent malignancies rather than differences in diagnostic surveillance, residual misclassification or shared exposure to tobacco and alcohol.

The timing of these diagnoses supports this interpretation. Among diagnoses at anatomically adjacent sites, 38.3% were documented within the first year after the landmark date, compared with 16.4% at other sites. This early concentration is compatible with residual capture of recurrent or persistent disease despite the 12-month landmark period, although timing alone cannot reliably distinguish recurrence from a true second primary tumor in routine primary care data [11,12].

From a clinical perspective, the findings support continued awareness of subsequent malignancies during long-term follow-up after oral cavity cancer, particularly within the upper aerodigestive and respiratory tracts. However, the present data do not provide a basis for specific screening strategies or surveillance intervals.

### 4.5. Strengths and Limitations

The main strengths of this study include its large sample size, the use of routine primary care data from more than 1000 practices in Germany, and the use of a matched comparison cohort rather than the general population. Matching accounted for age, sex, index year, comorbidity and consultation frequency before the index date. In addition, the 12-month landmark design reduced the influence of synchronous malignancies and diagnoses arising during the initial diagnostic work-up of the index tumor. The pre-specified sensitivity analysis and the analysis of event timing further allowed the potential contribution of anatomically ambiguous cancer codes to be assessed explicitly rather than assumed, and the results were consistent across a range of further sensitivity analyses.

Several limitations should be considered. First, cancer diagnoses were based on ICD-10 codes documented by general practitioners and were not verified using pathology reports or cancer registry data [31]. The recorded date may therefore reflect documentation in primary care rather than the actual date of cancer diagnosis, and misclassification is possible at all anatomical sites. Consequently, the 12-month landmark may not correspond exactly to 12 months after the actual diagnosis or treatment of the index tumor; however, restricting the analysis to patients with at least three years of prior observation or extending the landmark to 24 months yielded similar results (HR 2.41 and 2.40). In particular, recurrence, regional extension and true second primary malignancies could not be reliably distinguished for anatomically adjacent sites. Second, reliable dates of death were unavailable. Death could therefore not be incorporated as a competing event [32,33], and patients were censored at their last documented practice visit. Consequently, the Kaplan–Meier estimates may overestimate the real-world probability of a subsequent cancer diagnosis, particularly in the oral cavity cancer cohort, in which mortality is likely to be higher. Differential mortality may consequently have reduced the opportunity for patients with oral cavity cancer to receive a subsequent cancer diagnosis. Conversely, patients with a previous cancer diagnosis may undergo more intensive medical surveillance, as reflected by their higher visit frequency during follow-up, potentially increasing the likelihood and advancing the timing of documentation of additional malignancies [28]. The 12-month landmark period reduces but does not eliminate these opposing sources of bias. Third, information on tumor stage, histological characteristics, treatment, human papillomavirus status, smoking and alcohol consumption was unavailable. Matching could therefore account only for measured covariates, and residual confounding by unmeasured factors cannot be excluded. Fourth, individuals with an oral cavity cancer documented at any time were not eligible for the comparison cohort; including them with censoring at the diagnosis gave similar results (HR 2.35). Fifth, the CCI used for matching included diagnoses documented during the first year after the index date, which may reflect increased diagnostic attention or consequences of treatment. However, the mean CCI increased similarly in both cohorts during this year (by 0.36 and 0.38 points), and matching on a CCI based on diagnoses before the index date gave similar results (HR 2.41). Finally, the landmark design restricted the analysis to individuals who remained under observation beyond 12 months and had no additional cancer diagnosis documented during the first year. This excluded approximately one third of otherwise eligible patients with oral cavity cancer and limits the generalizability of the findings to patients who survived, remained in primary care and were free of another documented cancer during this initial period.

## 5. Conclusions

In German general practices, patients with documented oral cavity cancer had a higher incidence of subsequently documented cancer diagnoses than matched individuals without oral cavity cancer. Subsequent diagnoses involving the upper aerodigestive and respiratory tracts were disproportionately represented in the oral cavity cancer cohort. Excluding anatomically adjacent and potentially ambiguous cancer codes attenuated the association, but it persisted (HR 1.81). The available data do not establish whether these diagnoses represent confirmed second primary malignancies. The results describe an association between documented oral cavity cancer and subsequent cancer diagnoses and do not establish a causal relationship.

## Figures and Tables

**Table 1:** Baseline characteristics of the study sample after 1:10 propensity score matching.

Variable	Individuals with Oral Cavity Cancer (N = 1043)	Individuals Without Oral Cavity Cancer (N = 10,430)	SMD
Age (years), mean (SD)	63.5 (13.3)	63.6 (13.5)	0.005
Age ≤ 60 years	419 (40.2)	4188 (40.2)	<0.001
Age 61–70 years	325 (31.2)	3164 (30.3)	0.018
Age > 70 years	299 (28.7)	3078 (29.5)	0.019
Women	397 (38.1)	4047 (38.8)	0.015
Men	646 (61.9)	6383 (61.2)	0.015
Charlson Comorbidity Index, median (IQR)	2 (1–4)	2 (1–3)	0.022
CCI 0	178 (17.1)	1778 (17.0)	0.001
CCI 1	256 (24.5)	2573 (24.7)	0.003
CCI 2	196 (18.8)	1976 (18.9)	0.004
CCI 3	150 (14.4)	1518 (14.6)	0.005
CCI ≥ 4	263 (25.2)	2585 (24.8)	0.010
Index year 2005–2012	200 (19.2)	1983 (19.0)	0.004
Index year 2013–2016	207 (19.8)	2009 (19.3)	0.015
Index year 2017–2020	253 (24.3)	2547 (24.4)	0.004
Index year 2021–2024	383 (36.7)	3891 (37.3)	0.012
Practice visits in the 12 months before the index date, median (IQR) a	5 (1–11)	5 (1–11)	0.079

Data are *n* (%) unless otherwise indicated. SD, standard deviation; IQR, interquartile range; SMD, standardized mean difference; CCI, Charlson Comorbidity Index. a Number of days with a practice visit in the 12 months before the index date; the untransformed variable was used in the propensity score model. During follow-up, the median number of practice visits per year was 9.6 (IQR 4.2–16.8) in the oral cavity cancer cohort and 6.3 (IQR 2.2–11.9) in the matched cohort.

**Table 2:** Association between oral cavity cancer and a subsequent cancer diagnosis, overall and by age group and sex (Cox regression models).

Sub-Cohort	Incidence Per 1000 Person-Years (Events/Person-Years), Oral Cavity Cancer	Incidence Per 1000 Person-Years (Events/Person-Years), Without Oral Cavity Cancer	HR (95% CI)	*p*-Value
Total	38.0 (175/4609)	15.6 (722/46,337)	2.43 (2.06–2.87)	<0.001
Age ≤ 60 years	31.4 (68/2167)	7.6 (162/21,284)	4.07 (3.00–5.51)	<0.001
Age 61–70 years	46.7 (63/1349)	18.4 (258/14,018)	2.57 (1.98–3.33)	<0.001
Age > 70 years	40.2 (44/1093)	27.4 (302/11,036)	1.48 (1.08–2.03)	0.015
Women	28.3 (54/1905)	13.2 (236/17,876)	2.13 (1.61–2.82)	<0.001
Men	44.7 (121/2704)	17.1 (486/28,461)	2.62 (2.13–3.22)	<0.001

HR, hazard ratio; CI, confidence interval. Reference group: matched individuals without oral cavity cancer. Incidence rates and Cox models are based on the entire follow-up; standard errors account for the matched sets. *p* for interaction: age group < 0.001; sex 0.24.

**Table 3:** Association between oral cavity cancer and a subsequent cancer diagnosis by site of the oral cavity cancer (Cox regression models).

Oral Cavity Cancer Site	Incidence Per 1000 Person-Years (Events/Person-Years), Oral Cavity Cancer	Incidence Per 1000 Person-Years (Events/Person-Years), Without Oral Cavity Cancer	HR (95% CI)	*p*-Value
Tongue, other and unspecified parts (C02), *n* = 374	34.9 (56/1605)	16.5 (262/15,843)	2.11 (1.60–2.80)	<0.001
Gum (C03), *n* = 48	42.6 (10/235)	19.2 (48/2497)	2.28 (1.11–4.69)	0.024
Floor of mouth (C04), *n* = 318	37.5 (58/1548)	13.6 (212/15,604)	2.76 (2.05–3.70)	<0.001
Palate (C05), *n* = 76	33.2 (13/391)	17.5 (62/3542)	1.96 (1.07–3.61)	0.030
Other and unspecified parts of mouth (C06), *n* = 227	45.8 (38/830)	15.6 (138/8851)	2.95 (2.07–4.22)	<0.001

HR, hazard ratio; CI, confidence interval. Each site sub-cohort comprises the patients with the respective oral cavity cancer site and their matched individuals without oral cavity cancer. These analyses were exploratory and were not included in the Bonferroni correction. Standard errors account for the matched sets.

**Table 4:** Distribution of the cancer diagnoses documented during the entire follow-up, among patients in whom a cancer diagnosis was documented.

Site Group (ICD-10)	Oral Cavity Cancer Cohort (*n* = 175)	Cohort Without Oral Cavity Cancer (*n* = 722)
Lip, oral cavity and pharynx (C00–C14)	39 (22.3)	<10
Digestive organs (C15–C26)	25 (14.3)	128 (17.7)
Respiratory and intrathoracic organs (C30–C39)	31 (17.7)	77 (10.7)
of which bronchus and lung (C34)	26 (14.9)	69 (9.6)
Bone and articular cartilage (C40–C41)	<10	<10
Melanoma of skin (C43)	<10	23 (3.2)
Other malignant neoplasms of skin (C44)	24 (13.7)	130 (18.0)
Mesothelial and soft tissue (C45–C49)	<10	10 (1.4)
Breast (C50)	<10	59 (8.2)
Female genital organs (C51–C58)	<10	16 (2.2)
Male genital organs (C60–C63)	15 (8.6)	124 (17.2)
Urinary tract (C64–C68)	11 (6.3)	68 (9.4)
Eye, brain and central nervous system (C69–C72)	<10	10 (1.4)
Thyroid and other endocrine glands (C73–C75)	<10	<10
Other and ill-defined sites (C76)	<10	<10
Lymphoid and hematopoietic tissue (C81–C96)	<10	58 (8.0)
Multiple independent primary sites (C97)	<10	<10

Data are *n* (%) of all cancer diagnoses documented in the respective cohort; the percentages describe the distribution of sites and are not site-specific incidence estimates. The outcome definition was identical in both cohorts (C00–C97, excluding C02–C06 and C77–C80). Counts below 10 are not reported. Percentages may not sum to 100 because of rounding.

## Data Availability

The data used in this study are subject to strict privacy and confidentiality requirements and therefore cannot be made publicly available. Legal restrictions prohibit the sharing of the raw data with third parties.

## Supplementary Materials

The following supporting information can be downloaded at: https://www.mdpi.com/article/10.3390/cancers18213444/s1, Table S1: Sensitivity analysis and exploratory site-specific analyses (Cox regression models); Table S2: Further sensitivity analyses; Table S3: Assessment of the proportional hazards assumption; Table S4: Follow-up and loss to observation; Table S5: Distribution of the four-digit ICD-10 codes of the index tumor.

## Abbreviations

The following abbreviations are used in this manuscript:

CCI	Charlson Comorbidity Index
CI	Confidence interval
GDPR	General Data Protection Regulation
HR	Hazard ratio
ICD-10	International Classification of Diseases, 10th Revision
IQR	Interquartile range
SD	Standard deviation
SMD	Standardized mean difference
