# Supplementary material for: Oral Cavity Cancer and the Subsequent Incidence of Other Cancer Diagnoses: A Retrospective Cohort Study in German General Practices
Source: Cancers (Basel). 2026 Oct 24;18(21):3444. doi: 10.3390/cancers18213444 (PMC13762398; doi:10.3390/cancers18213444)
Supplement: Supplementary file 1 [file cancers-18-03444-s001.zip › cancers-4637762-supplementary.pdf]

Supplementary Materials

Oral cavity cancer and the subsequent incidence of other cancer diagnoses: a retrospective cohort study in German general practices

Table S1. Sensitivity analysis and exploratory site-specific analyses (Cox regression models)

Analysis	Sub-cohort	HR (95% CI)	p-value
Sensitivity analysis excluding C00–C14, C41 and C76 from the outcome	Total	1.81 (1.51–2.17)	<0.001
	Age ≤ 60	2.51 (1.76–3.59)	<0.001
	Age 61–70	2.15 (1.63–2.84)	<0.001
	Age > 70	1.20 (0.85–1.68)	0.299
	Women	1.73 (1.28–2.32)	<0.001
	Men	1.87 (1.48–2.35)	<0.001
Tongue (C02)	Age ≤ 60	4.41 (2.68–7.28)	<0.001
	Age 61–70	2.11 (1.37–3.26)	<0.001
	Age > 70	1.01 (0.55–1.86)	0.985
	Women	2.11 (1.42–3.12)	<0.001
	Men	2.13 (1.44–3.15)	<0.001
Gum (C03)	Age ≤ 60	4.75 (1.21–18.62)	0.025
	Age 61–70	not estimable	–
	Age > 70	2.99 (1.26–7.10)	0.013
	Women	2.39 (0.61–9.32)	0.210
	Men	2.44 (1.06–5.59)	0.035
Floor of mouth (C04)	Age ≤ 60	3.49 (1.99–6.12)	<0.001
	Age 61–70	3.32 (2.22–4.97)	<0.001
	Age > 70	1.69 (0.89–3.22)	0.108
	Women	2.00 (1.08–3.70)	0.028
	Men	3.10 (2.21–4.34)	<0.001
Palate (C05)	Age ≤ 60	2.93 (1.21–7.10)	0.017
	Age 61–70	2.34 (0.82–6.66)	0.110
	Age > 70	0.45 (0.06–3.74)	0.464
	Women	2.87 (1.34–6.14)	0.007
	Men	1.34 (0.50–3.59)	0.563
Other/unspecified mouth (C06)	Age ≤ 60	6.00 (2.92–12.33)	<0.001
	Age 61–70	3.18 (1.67–6.06)	<0.001
	Age > 70	1.94 (1.14–3.31)	0.015
	Women	2.01 (0.91–4.41)	0.084
	Men	3.39 (2.27–5.06)	<0.001

HR, hazard ratio; CI, confidence interval. Standard errors account for the matched sets. The sensitivity analysis additionally excludes C00–C14, C41 and C76 from the outcome definition in both cohorts, using the same matched cohort as the primary analysis (p for interaction: age group 0.005; sex 0.69). The site-specific models use

the primary outcome definition. The model for gum cancer in the age group 61–70 years could not be estimated because no cancer diagnosis was documented in the oral cavity cancer group. None of these analyses were included in the Bonferroni correction.

Table S2. Further sensitivity analyses (Cox regression models)

Analysis	N, OCC / comparison	Events, OCC / comparison	Incidence per 1,000 PY, OCC / comparison	HR (95% CI), primary outcome	HR (95% CI), sensitivity outcome
Primary analysis (matching on practice visits in the 12 months before the index date)	1,043 / 10,430	175 / 722	38.0 / 15.6	2.43 (2.06–2.87)	1.81 (1.51–2.17)
Outcome definition excluding only C02–C06 (and C77–C80) in both cohorts	1,043 / 10,430	175 / 722	38.0 / 15.6	2.43 (2.06–2.87)	–
Matching without visit frequency	1,043 / 10,430	175 / 731	38.0 / 16.2	2.35 (1.99–2.77)	1.75 (1.45–2.10)
Matching on visit frequency during follow-up	1,043 / 10,430	175 / 803	38.0 / 17.2	2.21 (1.88–2.60)	1.65 (1.37–1.98)
Individuals with a later oral cavity cancer eligible for the comparison cohort, censored at that diagnosis	1,043 / 10,430	175 / 746	38.0 / 16.1	2.35 (2.00–2.77)	1.75 (1.46–2.09)
Matching on a Charlson Comorbidity Index calculated from diagnoses documented before the index date	1,043 / 10,430	175 / 718	38.0 / 15.8	2.41 (2.04–2.84)	1.80 (1.50–2.17)
Robust variance clustered by general practice	1,043 / 10,430	175 / 722	38.0 / 15.6	2.43 (2.08–2.85)	1.81 (1.51–2.17)
Cox model stratified by matched set	1,043 / 10,430	175 / 722	38.0 / 15.6	2.64 (2.19–3.18)	1.99 (1.63–2.44)
Matched sets of patients with at least 3 years of observation before the index date	841 / 8,410	142 / 593	39.0 / 16.2	2.41 (2.01–2.89)	1.76 (1.44–2.16)
Landmark 24 months after the index date	791 / 8,177	136 / 567	36.8 / 15.3	2.40 (2.00–2.88)	1.90 (1.55–2.32)
Follow-up truncated at 10 years	1,043 / 10,430	157 / 648	37.3 / 15.3	2.44 (2.04–2.91)	1.84 (1.52–2.23)
Matched sets excluding index codes C02.4, C05.1 and C05.2	1,017 / 10,170	170 / 700	38.0 / 15.6	2.44 (2.06–2.89)	1.79 (1.49–2.16)
Lung cancer (C34) as outcome	1,043 / 10,430	31 / 79	6.1 / 1.6	3.74 (2.43–5.76)	–

OCC, oral cavity cancer; PY, person-years; HR, hazard ratio; CI, confidence interval. Events and incidence rates refer to the primary outcome and the entire follow-up (except for the truncated analysis). The sensitivity outcome additionally excludes C00–C14, C41 and C76. Standard errors account for the matched sets unless stated otherwise. In the 24-month landmark analysis, individuals with a cancer diagnosis or the end of observation during the second year after the index date were excluded and follow-up started 24 months after the index date. In the primary analysis, individuals with a documented oral cavity cancer were not eligible for the comparison cohort, so that the definition excluding only C02–C06 gives identical results. All p-values < 0.001.

Table S3. Assessment of the proportional hazards assumption

Outcome	Schoenfeld residuals, correlation with rank of time (p)	Exposure × log(time), HR (95% CI); p	HR (95% CI), 0–1 year	HR (95% CI), >1–3 years	HR (95% CI), >3 years
Primary outcome	0.008 (0.81)	1.01 (0.89–1.15); 0.87	2.56 (1.78–3.66)	2.33 (1.65–3.28)	2.43 (1.95–3.04)
Sensitivity outcome	0.027 (0.43)	1.07 (0.92–1.24); 0.40	1.46 (0.93–2.28)	1.98 (1.37–2.86)	1.87 (1.46–2.38)

HR, hazard ratio; CI, confidence interval. Time refers to time since the landmark date. Period-specific HRs were estimated with time-dependent exposure indicators in one model; standard errors account for the matched sets. Number of events in the oral cavity cancer and comparison cohorts (primary outcome): 39 and 155 (0–1 year), 40 and 177 (>1–3 years), 96 and 390 (>3 years).

Table S4. Follow-up and loss to observation in the matched cohorts

Characteristic	Oral cavity cancer cohort (N = 1,043)	Comparison cohort (N = 10,430)
Median follow-up, years (IQR)	2.9 (1.1–6.9)	3.0 (1.2–6.6)
Person-years of follow-up	4,609	46,337
Cancer diagnosis documented, n (%)	175 (16.8)	722 (6.9)
Last documented visit more than 12 months before the end of the database, without cancer diagnosis, n (%)	335 (32.1)	3,600 (34.5)
Last documented visit within 12 months before the end of the database, without cancer diagnosis, n (%)	533 (51.1)	6,108 (58.6)
Still under observation after 1 year, %	79.1	79.7
Still under observation after 3 years, %	54.6	52.7
Still under observation after 5 years, %	41.5	36.9
Still under observation after 10 years, %	17.3	13.7
Practice visits per year during follow-up, median (IQR)	9.6 (4.2–16.8)	6.3 (2.2–11.9)

IQR, interquartile range. End of the database: February 2026. The proportions still under observation were estimated with the reverse Kaplan–Meier method, treating the end of observation without a documented cancer diagnosis as the event. Reasons for leaving observation (death, change of practice, relocation) cannot be distinguished in the database.

Table S5. Distribution of the four-digit ICD-10 codes of the index tumor in the oral cavity cancer cohort (N = 1,043)

ICD-10 code	Description	n (%)
C02.0	Dorsal surface of tongue	32 (3.1)
C02.1	Border of tongue	74 (7.1)
C02.2	Ventral surface of tongue	13 (1.2)
C02.3	Anterior two-thirds of tongue, part unspecified	15 (1.4)
C02.4	Lingual tonsil	<10
C02.8	Overlapping lesion of tongue	<10
C02.9	Tongue, unspecified	231 (22.1)
C03.0	Upper gum	17 (1.6)
C03.1	Lower gum	27 (2.6)
C03.9	Gum, unspecified	<10
C04.0	Anterior floor of mouth	29 (2.8)
C04.1	Lateral floor of mouth	14 (1.3)
C04.8	Overlapping lesion of floor of mouth	20 (1.9)
C04.9	Floor of mouth, unspecified	255 (24.4)
C05.0	Hard palate	<10
C05.1	Soft palate	12 (1.2)
C05.2	Uvula	13 (1.2)
C05.8	Overlapping lesion of palate	<10
C05.9	Palate, unspecified	39 (3.7)
C06.0	Cheek mucosa	78 (7.5)
C06.1	Vestibule of mouth	<10
C06.2	Retromolar area	<10
C06.8	Overlapping lesion of other and unspecified parts of mouth	<10
C06.9	Mouth, unspecified	143 (13.7)

Counts below 10 are not reported. In 20 patients, two different oral cavity sites (three-digit codes) were documented on the index date; the first recorded code determined the index site.
